# Attrition of amino acids in the life span of broiler chickens

**DOI:** 10.1016/j.aninu.2025.05.004

**Published:** 2025-07-12

**Authors:** Shemil P. Macelline, Sonia Y. Liu, Peter H. Selle

**Affiliations:** a School of Life and Environmental Sciences, Faculty of Science, The University of Sydney, Camperdown 2006, NSW, Australia; b Poultry Research Foundation, The University of Sydney, Camden 2570, NSW, Australia; c Sydney School of Veterinary Science, The University of Sydney, Camperdown 2006, NSW, Australia

**Keywords:** Amino acid metabolism, Broiler chicken, Digestive dynamics, Protein turnover

## Abstract

A dietary protein intake of approximately 2.5 kg is required to generate 1.0 kg of protein in the carcass of broiler chickens at 42 d post–hatch, reflecting the attrition or wastage of dietary amino acids that occurs during their lifespan. Thus, the aim of this review is to examine amino acid utilisation in broilers because the identification and correction of any avoidable losses will advantage both the chicken meat industry and consumers. Amino acids are lost to catabolic pathways in their transition across the enterocytes for energy production required for digestive processes. However, slowly digestible starch has been indicated to spare amino acids from catabolic losses by providing more glucose as an alternative energy substrate. This potential benefit warrants further investigation. Protein turnover may represent an even greater source of amino acid attrition, but it is a complex area. Protein turnover refers to the dynamic equilibrium between protein deposition and degradation, with a positive balance indicating net protein synthesis and growth. 3-Methylhistidine concentrations in systemic plasma may be a sufficiently accurate indicator of protein degradation, which is probably the more influential component in protein turnover. If this can be demonstrated, experiments involving both L-carnitine and 3-methylhistidine may prove highly instructive because there are indications that L-carnitine has the capacity to depress protein degradation and enhance protein turnover in broiler chickens. The function of insulin in avian species is essentially a conundrum, particularly in relation to its regulatory role over protein turnover as broilers mature, and, ideally, this should be elucidated. Importantly, nutritionists should continue to endeavour to formulate diets for broiler chickens that meet amino acid requirements accurately. When this is achieved, circulating ammonia levels arising from post-enteral amino acid imbalances will be limited to the advantage of protein turnover and broiler growth performance. It is possible that protein degradation would be attenuated by broiler diets with appropriate balances between protein-bound and non-bound entities, ideal amino acid ratios and higher dietary electrolyte balances.

## 1 Introduction

Chicken-meat benefits from a strong global consumer demand, whether purchased as an inexpensive, healthy or environmentally friendly option (Clements, 2024). Poultry, as a share of total meat consumption has been growing for decades and it is expected to account for 43% of meat consumed from all sources by 2033 based on OECD-FAO (2024) forecasts. These forecasts predict that consumption of poultry meat globally will be 16% higher by 2033, when it is projected to reach the equivalent of 160 million metric tonnes retail weight. The prime reasons driving poultry's share of total meat consumption is simply because it is the lowest priced meat available in the marketplace (Clements, 2024) and chicken-meat is accepted by consumers of all cultures. This all-important price advantage stems mainly from the tremendous advances achieved by the geneticists in the selection of broiler chickens over decades, as documented by Zuidohf et al. (2014). The 2022 weight gain objectives for Ross 308 mixed-sex birds (Aviagen, 2022) may be compared with the data for 1978 birds of Zuidohf et al. (2014). The modern bird enjoys weight advantages of 187% (1816 vs. 632 g/bird) at 28 d and 139% (4318 vs.1808 g/bird) at 56 d post–hatch. By any benchmark, the progress realised over 44 years in broiler growth performance is extraordinary.

The outlook for the future demand for chicken-meat appears buoyant; however, this will be contingent on maintaining current relative price advantages. Thus, the challenge will be to contain production costs, which essentially equate to feed costs, as feed costs represent up to 70% of total production costs (Thirumalaisamy et al., 2019). Soybean meal is the principal protein source and maize, coupled with wheat, are the major sources of starch and energy in broiler diets globally. Soybeans are mostly grown in North and South America, which means that soybean meal is an expensive, imported feedstuff for most countries in the world. According to one forecast, the global soybean meal market will increase from US$59.2 billion in 2021 to US$94.2 billion in 2031 (Allied Market, 2023), which suggests the continued usage of soybean meal by the global chicken meat industry at present levels is not sustainable. Consequently, there is a pressing need to identify alternative protein sources, where it appears that canola-derived proteins may be promising substitutes as recently reviewed by Kandel et al. (2025). Moreover, scope does appear to exist to enhance the nutritive value of canola meals, in particular, for broiler chickens by improved processing methods. Alternatively, the successful development and adoption of reduced-crude protein (CP) diets for broiler chickens would also diminish the demand for soybean meal (Greenhalgh et al., 2020a). As one example, dietary CP concentrations were reduced in a step-wise manner from 200 to 188, 172 and 156 g/kg in maize-based diets formulated to contain 11.0 g/kg digestible lysine with a metabolisable energy density of 12.85 MJ/kg in Chrystal et al. (2020). Following the CP reduction from 200 to 156 g/kg, soybean meal inclusions decreased by 48.0% (171 vs. 329 g/kg), which is substantial. To counter this, maize increased by 22.1% (719 vs. 560 g/kg) and non-bound amino acids (NBAA) by a 4.3-fold factor (24.86 vs. 5.81 g/kg) to meet specifications.

The potential to enhance the conversion of dietary protein into skeletal muscle protein in broiler chickens is a highly attractive objective, but involves numerous, complex physiological processes. Optimising amino acid utilisation for muscle protein synthesis aligns with the principles of sustainable chicken meat production. For example, reducing dietary-CP concentrations by 50 g/kg without compromising broiler growth performance has been estimated to enhance the dietary protein-to-carcass protein ratio from 2.51 to 1.89 (Macelline et al., 2021). Dietary-CP reductions in broiler diets alter the digestive dynamics of amino acids compared to conventional CP diets (Ibrahim et al., 2024a, Ibrahim et al., 2024b; Liu et al., 2021a, Liu et al., 2021b; Macelline et al., 2023c). These reductions necessitate higher inclusions of NBAA, which differ from protein-bound amino acids in their rates of intestinal uptakes (Liu et al., 2021a, Liu et al., 2021b; Krehbiel and Matthews, 2003). Selle et al. (2022b) specifically addressed the lack of bioequivalence between NBAA and PBAA. Reduced-CP diets typically have elevated dietary starch concentrations, which can influence amino acid digestibilities (Moss et al., 2018). Furthermore, starch digestion rates of feed grains may influence the oxidation of amino acids in avian enterocytes which imposes a metabolic cost and amino acid attrition (Luo et al., 2025; Weurding et al., 2003; Yin et al., 2019).

Proteins, and their constituent amino acids, are invaluable feedstuffs as the rapid growth rates achieved by modern broilers are largely a result of net protein synthesis in skeletal muscle. Breast, tenderloins, wings and leg quarters represent 68.6% (1477 of 2152 g) of the carcass weight of birds at 42 d post–hatch (Weimer et al., 2022). Broiler chickens utilise protein and amino acids far more efficiently than other terrestrial food producing animals (Wu et al., 2014); nevertheless, Macelline et al. (2021) estimated that an intake of 2.51 kg of dietary protein is required to generate 1.00 kg of protein in the carcass of a broiler chicken at 42 d post–hatch. This ratio suggests that improvements can be realised; thus, the objective of this review is to examine the overall utilisation of amino acids in broilers. Clearly, the identification and correction of any avoidable losses, or attrition, of amino acids during the life span of broilers will only benefit the chicken meat industry and consumers alike.

## 2 Intestinal uptakes of amino acids

A standard broiler diet contains about 210 g/kg CP, predominantly as intact protein; however, a tangible proportion of this protein fails to enter the portal circulation and systemic pool as amino acids. This is a culmination of incomplete protein digestibility and absorption of amino acids, fermentation of amino acids along the gastrointestinal tract, particularly in the hind-gut, and the capture of amino acids by anabolic and catabolic pathways in enterocytes of the small intestinal mucosa. Intestinal uptakes of amino acids are dependent upon both digestion of intact protein in the gut lumen and the absorption of amino acids into enterocytes along the small intestine. The likelihood is that absorption of amino acids is the more limiting factor on the growth performance of broiler chickens than the digestion of protein (Croom et al., 1999). Finally, the passage of amino acids throughout the gut mucosa and their partial utilisation by enterocytes and the portal drained viscera determines their proportionate entry into the portal and systemic circulations (Stoll et al., 1999). However, our comprehension of the intestinal uptakes of amino acids and their utilisation in the enterocytes in broiler chickens is not complete.

### 2.1 Digestion of intact protein

The enzymatic digestion of intact dietary protein by proteolytic endogenous enzymes throughout the small intestine is well-established, with protein digestion in poultry specifically reviewed by Noy and Sklan (1995) and Moran (2016). Briefly, the process begins in the proventriculus, where proteins are denatured by the low pH created by HCl secretions, facilitating their breakdown by pepsin. The peptide fragments produced by pepsin digestion stimulate the release of cholecystokinin and gastrin, which play regulatory roles in protein digestion (Krehbiel and Matthews, 2003). In the duodenum, various pancreatic proteolytic enzymes, including trypsin, chymotrypsin, carboxypeptidase, and elastase are secreted to break down polypeptides into smaller peptide fragments. These fragments are further hydrolyzed into di- and tripeptides (or oligopeptides) by aminopeptidases and dipeptidases located on the apical membrane of enterocytes (Moran, 2016).

The phenomenon of reverse peristalsis, which involves the recycling of digesta within the gastrointestinal tract, distinctly sets avian species apart from mammals. The retention time of digesta along the gastrointestinal tract in broiler chickens is brief, which is a challenge to both digestion and absorption of nutrients. Shires et al. (1978) estimated retention times of 62 min in the crop, proventriculus and gizzard, 153 min in the small intestine and 123 min in the large intestine for a total retention time of 338 min. However, episodes of reverse peristalsis add complexity to digesta retention times throughout the digestive tract (Duke, 1982). Reverse peristalsis occurs in three distinct regions in the gut: (1) the gizzard, (2) the small intestine, and (3) the cloaca-caecum (Ferket, 2000). Gastric reflux recycles digesta from the gizzard back to the proventriculus through gastro-duodenal contractions, while small intestinal reflux returns chyme from the duodenum and jejunum to the gastric area. The gizzard is considered the pacemaker of gut motility, plays a key role in regulating episodes of reverse peristalsis (Ferket, 2000). Additionally, whole grain feeding, which promotes the development of heavier and more functional gizzards, is likely to extend digesta retention in the gizzard (Liu et al., 2014). Increased episodes of reverse peristalsis should facilitate protein and starch digestion in the gut lumen. Inert dietary markers, such as titanium dioxide or acid insoluble ash, are commonly utilized to assess amino acid digestibility in broiler digestibility experiments. However, because of the retention and recycling of digesta, discrepancies may arise in the passage rates of the inert marker and other digesta components. Furthermore, the passage rate of solids through the gizzard is approximately 40 min, while liquid pass about in just 17 min (Sklan et al., 1978). This suggests that water soluble, non-bound amino acids with low molecular weights may separate or leach into the liquid phase of the digesta retained in the crop and gizzard, potentially not being recycled as efficiently via reverse peristalsis. Additionally, broiler chickens can store substantial quantities of feed in their crops, and in preparation for scotoperiods, or periods of darkness, birds may ‘crop-up’ and retain digesta (Classen et al., 2016).

### 2.2 Absorption of amino acids

The majority of amino acids in standard diets, perhaps 75%, are absorbed as di- and tri-peptides rather than monomeric amino acids (Matthews and Adibi, 1976). Their intestinal uptakes are via the oligopeptide transporter PepT-1 functioning in tandem with the Na^+^/H^+^ sodium-hydrogen exchanger NHE as the function of PepT-1 requires proton donations (Daniel, 2004). In contrast, NBAA, including supplementary amino acids, are absorbed through a range of Na^+^-dependent and Na^+^-independent transport systems, which have overlapping affinities (Hyde et al., 2003). The NBAAs are considered to be 100% digestible as they do not involve digestion (Lemme et al., 2005), but intestinal uptakes of di- and tri-peptides are considered to be more efficient that monomeric amino acids (Matthews, 1972). Interestingly, Eugenio et al. (2023) determined the apparent jejunal digestibility coefficients of 18 amino acids in sacrificed pigs using two digestibility markers. Pigs offered diets containing intact protein (170 g/kg feather meal) had a mean digestibility coefficient of 0.773. When the feather meal was extensively hydrolysed the mean digestibility coefficient increased substantially to 0.949. Alternatively, pigs provided equivalent diets containing only ‘free’ amino acids (NBAA) had a 4.00% lower mean digestibility coefficient of 0.911, with remarkably low coefficients for histidine (0.701) and cysteine (0.833). This suggests that, in this context, absorption of free amino acids via relevant transport systems can become limiting and their intestinal uptakes compromised. Digestibility coefficients assess both digestion and absorption and the configuration of an amino acid in the gut lumen clearly has a tangible impact on its intestinal uptake. It is unsurprising that shifting from standard to reduced-CP diets leads to disruptions in amino acid digestibility coefficients in broiler chickens. This was highlighted in the Liu et al., 2021a, Liu et al., 2021b review, which examined the effects of dietary-CP reductions on apparent ileal amino acid digestibility coefficients across seven studies, where substantial perturbations in amino acid digestibility were reported.

Vinardell (1990) documented the mutual inhibition between sugars and amino acids during intestinal absorption. So, another consideration is that digestibility coefficients of starch were negatively related to digestibility coefficients of eight amino acids (arginine, histidine, isoleucine, methionine, phenylalanine threonine, valine, glutamate) in the three small intestinal segments (distal jejunum, proximal and distal ileum) in Moss et al. (2018), where broiler chickens were offered reduced-CP diets. This was attributed to competition between glucose and amino acids for co-absorption with sodium for intestinal uptakes via their respective Na^+^-dependent transport systems. Intestinal uptakes of glucose are principally via SGLT-1, a Na^+^-dependent transporter (Shibata et al., 2019), but non-bound amino acids are also mainly absorbed via Na^+^-dependent transporters (Miska and Fetterer, 2019).

### 2.3 Transition of amino acids across gut mucosa

Our understanding of the metabolic fates of amino acids in the enterocytes of the small intestinal gut mucosa in avian species is inadequate. The process by which oligopeptides and amino acids absorbed along the small intestine enter the systemic circulation is complex, as amino acids may be redirected into anabolic or catabolic pathways within enterocytes, preventing their entry into the portal circulation.

In anabolic pathways, amino acids can be converted into proteins that help conserve gut integrity or/and be used for production of mucin, digestive enzymes, non-essential amino acids polyamines, and nucleotides (Wu, 1998). The intrinsic flow of endogenous amino acid in broiler chickens demonstrate these anabolic pathways and include mucin, digestive enzymes, desquamated intestinal epithelial cells, and some microbial amino acids (Ravindran, 2021). Mucin serves as a key source of endogenous amino acids since it remains undigested, and its amino acids are not reabsorbed; for instance, porcine mucin contains a protein concentration of 343 g/kg (Lien et al., 1997). The amino acid constituency of mucin in avian species has been determined by Fang et al. (1993), in which threonine and serine are dominant.

The preferred energy substrates in catabolic pathways of the small intestinal enterocytes in broiler chickens have been identified as glucose, glutamate, glutamine, aspartate and asparagine (Porteous, 1980; Watford et al., 1979). The likelihood is that glutamate, a ubiquitous amino acid, provides the most energy to the enterocytes (He et al., 2021). In pigs, about 97% of dietary glutamate is oxidized to carbon dioxide by the enterocytes prior to passing into the portal vein (Hou and Wu, 2018). If this applies to birds, it follows that concentrations of glutamate (and glutamine) in systemic plasma must be largely derived from endogenous synthesis, the adequacy of which becomes questionable.

The energy demands of the gut for digestive processes are considerable (Cant et al., 1996). Reeds et al. (1999) emphasized that amino acids serve as vital energy sources for the intestinal mucosa but, importantly, raised the question of whether this could be influenced by nutritional strategies. The catabolism of glucose and glutamine provided similar proportions of energy to isolated rat enterocytes (Fleming et al., 1997), so it follows that the ‘catabolic ratio’ between glucose and amino acids within enterocytes may be manipulable by dietary strategies to benefit the host. In broiler diets, slowly digestible starch has been demonstrated to improve growth performance (Herwig et al., 2019). It has been suggested that this improvement stems from slowly digestible starch sparing amino acids catabolism in the broiler enterocytes (Enting et al., 2005; Weurding et al., 2003). It follows that slowly digestible starch would make more glucose available to enterocytes in the posterior small intestine, which would not apply to rapidly digestible starch. This relative lack of glucose would effectively force enterocytes in the caudal small intestine to catabolise amino acids for energy provision. It has been suggested that NBAA are less likely to be catabolised than PBAAs in enterocytes (Moss et al., 2018). This proposal was based on determinations of free amino acid plasma concentrations in blood taken from the anterior mesenteric vein. The researchers implied that NBAA might moderately evade catabolism in the gut mucosa, as their absorption occurs in the anterior small intestine, where glucose availability provides a complementary energy substrate.

## 3 Post-enteral amino acid metabolism

The likelihood is that protein turnover is pivotal to the post enteral amino acids metabolism. Protein turnover is the dynamic balance between deposition and degradation of whole-body protein and is of particular importance in skeletal muscle. However, protein turnover in broiler chickens has not been extensively investigated.

### 3.1 Role of the liver

Amino acids carried from the small intestine, via the portal vein, to the liver, which has been described as the ‘forgotten organ’ in poultry (Zaefarian et al., 2019). There is some hepatic protein synthesis and amino acids are carried to other tissues and organs via the systemic circulation. Importantly, amino acids exceeding requirements for protein synthesis and/or functional purposes are catabolised by the liver where hepatocytes deaminate amino acids to generate ammonia (NH_3_) and keto acids. Because NH_3_ is fundamentally toxic (Stern and Mozdziak, 2019), perhaps more so in avian compared to mammalian species (Wilson et al., 1968), NH_3_ requires detoxification. This is achieved by NH_3_ conversion into the uric acid by uric acid cycle, which is excreted via the urine. Detoxification of NH_3_ takes place mainly in the liver of birds with a minority, some 17%, occurring in the kidneys (Chin and Quebbemann, 1987). Concentrations of glutamine are relatively high in avian livers as glutamine is an intermediate in the synthesis of uric acid. Higher NH_3_ concentrations in plasma and NH_3_ detoxification in broiler chickens are considered in more depth in Section 4.4.

The metabolism of amino acids in the liver was thoroughly reviewed by Hou et al. (2020) with a focus on nutrition and physiology. The liver plays a vital role in the metabolism, transport and storage of nutrients and is also involved in detoxification. In mammals the liver degrades all amino acids, other than branched-chain amino acids, and is involved in the synthesis of non-essential amino acids and glucose. The mammalian liver also converts phenylalanine into tyrosine and methionine into cysteine. Degradation of amino acids mainly takes place in the liver; however, in pigs offered diets containing optimal amounts of amino acids, degradation was attenuated (Kristensen and Wu, 2012).

### 3.2 Protein turnover—skeletal muscle

Protein turnover is the continuous flux between deposition and degradation of whole-body protein, where a positive balance represents net protein synthesis and growth. Broilers presently exhibit increasingly express growth rates and male broiler chickens may weigh more than 3.25 kg at 42 d post–hatch. This rate of gain is largely driven by net protein synthesis of skeletal muscle, which, as breast, tenderloins, wings and leg quarters, represents 68.6% (1477 of 2152 g) of the carcass weight of birds at 42 d post–hatch (Weimer et al., 2022). The protein turnover rate in broiler chickens (30.2 vs. 16.1 g/kg body weight^0.75^/d) is nearly double the average rate of four mammalian species (mouse, rat, sheep, man), as tabulated by Muramatsu (1990). The rates of protein deposition and degradation exceed dietary protein intakes; consequently, free plasma amino acid concentrations are derived both endogenously, from protein turnover and biosynthesis of non-essential amino acids, and exogenously, from intestinal uptakes of amino acids (Reeds and Fuller, 1983).

Is noteworthy that Sunde et al. (1984) stressed the importance of protein degradation decades ago and these researchers emphasised the need to develop the capacity to quantify protein turnover. They also suggested that there should be more focus on protein degradation, rather than protein deposition, in the quest to enhance broiler growth performance. The following study by Tesseraud et al. (2000) supports the tenet that protein degradation is the more influential element in protein turnover. In this study muscle protein turnover in chickens selected for divergent growth rates were determined by a flooding dose of L-[4-^3^H] phenylalanine. Tesseraud et al. (2000) concluded that heavier weights of the pectoralis major muscle in rapidly growing birds were essentially a consequence of decreased protein degradation. The fractional protein breakdown in the fast-growing birds was 38.0% (10.3 vs. 16.6%/d) slower in comparison to the slow-growing birds at 14 d post–hatch. In contrast, the difference in fractional protein synthesis between the two lines was not significantly different. The underlying mechanisms have yet to be elucidated; however, the possibility that altered regulation of the ubiquitin-proteasome pathway difference between fast- and slow-growing birds was raised. Ubiquitin-proteasome pathway promoted protein degradation.

Protein degradation is intriguing because superficially it seems counter-intuitive, which led Reeds (1989) to question the rationale for the evolution of such a process and Rothman (2010) to question how the balance between protein deposition and degradation is achieved. Nevertheless, the essential role of protein degradation is to preserve the integrity of protein (Avci and Lemberg, 2015) as protein turnover serves to maintain protein homoeostasis. Proteostatic mechanisms ensure that functional proteins are held at their correct concentrations and in the proper locations required for cellular activities. They also ensure that misfolded, aged, or damaged proteins are removed whenever necessary (Ross et al., 2021).

The ubiquitin-proteasome pathway is the principal route via which intracellular proteins are degraded and this pathway has been described as “destruction for the sake of construction” (Glickman and Ciechanover, 2002). The ubiquitin-proteasome pathway links chains of the polypeptide co-factor, ubiquitin onto proteins to mark them for degradation (Goldberg, 2003). According to Schutz (2011), amino acids released by endogenous protein breakdown can be reconverted to protein with very little loss. However, Osana et al. (2022) recently contended that recycled-amino acids from proteasomal proteolysis are used primarily for energy (ATP) production rather than de novo protein synthesis. If this tenet is valid, protein degradation represents a real attrition of amino acids and should be minimised to the extent possible. In addition to the ubiquitin-proteasome pathway, some cytosolic proteins are degraded in lysosomes in a process accelerated by the lack of insulin or essential amino acids and in the liver by glucagon (Lecker et al. (2006). Somewhat surprisingly, there are few suggestions in the literature that whole body protein is degraded to meet amino acid deficiencies in specific organs, which would appear to be a reasonable assumption.

### 3.3 Insulin and protein turnover

Insulin is a powerful anabolic hormone and, in humans, insulin was considered critical in maintaining proteome homeostasis by James et al. (2017) as insulin signaling regulates both protein synthesis and degradation. Earlier, Fawcett et al. (2001) argued that the major effect of insulin on protein turnover was the inhibition of protein degradation in adult animals. The importance of insulin in broiler chickens is usually dismissed because it is generally accepted that avian species are hyperglycaemic and resistant to insulin (Braun and Sweazea, 2008; Sweazea, 2022). However, it appears that insulin does play an anabolic role in broilers up to about 21 d of age, which is not generally appreciated (Selle et al., 2025). Instructively, Tokushima et al. (2003) concluded that newly hatched chicks are under the control of specific insulin—glucose interactions characterised by low plasma insulin concentrations with high sensitivity to insulin. These researchers found that 60-min decreases in plasma glucose concentration following 10 and 40 mg/kg insulin injections were greater in 7-d-old chicks than in 21-d-old birds. A 40 μg/kg insulin injection reduced plasma glucose concentrations by approximately 54% in 7-d-old chicks as opposed to 39% in 21-d-old chicks 60 min post-administration. Similarly, insulin sensitivity was shown to be greater in the first two weeks than in the fourth week in White Leghorn chicks (Joseph et al., 1996). Indeed, insulin resistance appears to develop post–hatch, possibly because of perturbations in the insulin signalling cascade (Franssens et al., 2014). Thus, the insulin status is not a constant throughout the embryonic and post–hatch stages of the broiler lifecycle; a 42-d-old bird is probably resistant to insulin while a 7-d-old chick is probably sensitive to insulin. If valid, the obvious challenge that arises is the identification of the additional mechanisms that regulate protein turnover in older broilers, other than insulin and presumably glucagon.

It is noteworthy that glucose stimulated insulin and glucagon release from cultured avian pancreatic tissue taken from 15 to 18-d-old embryos and from 2 and 9-d-old chicks in Foltzer et al. (1982). In respect of insulin, a glucose concentration of 30 mM was the most effective and the responsiveness of the pancreatic tissue taken from chicks was less than that taken from embryos. Remarkably, a single administration of insulin (400 μg/kg) to 1-d-old chicks significantly increased body weights by 3.54% (935 vs. 903 g/bird) at 21 d and by 3.07% (3631 vs. 3523 g/bird) at 50 d post–hatch in Sato et al. (2012).

Bovine insulin decreased protein degradation in chicken muscle tissues taken from 16-d-old birds by an average of 10.8%, irrespective of the presence of glucose in the incubation medium, in an in vitro experiment conducted by Klasing and Jarrell (1985). This suggested the action of insulin on protein degradation was not secondary to effects on glucose transport. Klasing and Jarrell (1985) concluded that insulin decreases rates of protein degradation in chicken muscle; thus, this in vitro data is consistent with the proposal that insulin has an anabolic impact in skeletal muscles of (young) broiler chickens, which may have been more pronounced had avian insulin been used. The insulin anabolic signalling cascade in broiler chickens in connection with the effects of n-3 polyunsaturated fatty acids was investigated by Tesseraud et al. (2014). In this study 23-d-old, fasted birds were administered 6.9 mmol/kg (human) insulin intra-venously and blood samples were taken after 15 min. Insulin depressed average plasma glucose concentrations by 32.2% (1.45 vs. 2.14 g/L). This outcome suggests that broiler chickens are partially sensitive to insulin at 23 d of age. Again, it would have been preferable had avian insulin had been used in this study. The point is made by Dupont et al. (2009) that most insulin assays in broilers have not been completed with avian insulin, which could result in misleading outcomes.

Presently, as-hatched broiler chickens attain a target weight of 2.4 kg at 35 d post–hatch or less. Thus, the interval during which birds retain some insulin sensitivity during the post-natal, grow-out phase will proportionately increase given the continued selection for rapidly growing broilers.

### 3.4 Elevated plasma NH_3_ concentrations and NH_3_ detoxification

The likelihood is that reduced-CP broiler diets, especially when based on wheat, can trigger elevated plasma ammonia (NH_3_) concentrations or "NH_3_ overload" (Selle et al., 2022a, 2023a, 2023b). For example, male broiler chickens were fed 210, 190 and 170 g/kg CP wheat-based diets in which NBAA inclusions increased from 15.1 to 39.0 and 55.4 g/kg in Macelline et al. (2023a). As a result, plasma NH_3_ concentrations increased by up to 17.1%, from 1.87 to 2.04 and 2.19 μg/mL at 35 d post–hatch. The 40 g/kg dietary CP reduction reduced weight gains by 13.4% and breast muscle yields by 8.30%. Increasing plasma NH_3_ concentrations were negatively related with weight gains (*r* = −0.564; *P* = 0.018) and feed intakes (*r* = −0.487; *P* = 0.018). The second relationship is consistent with NH_3_ intoxication depressing voluntary feed intakes. Given the inherent toxicity of NH_3_ this outcome is not surprising and there are precedents for ammonia overload having deleterious impacts on broiler growth performance (Aguihe et al., 2022; Namroud et al., 2008; Ospina-Rojas et al., 2013, 2014).

It is likely that post-enteral disparities of PBAA and NBAA, along with the catabolism of excess amino acids, lead to elevated NH_3_ concentrations in broiler chickens (Selle et al., 2022b). This is because amino acids beyond the requirements for protein synthesis are rapidly catabolised (Brosnan, 2003). Consequently, NBAA are more prone to catabolism or post-prandial oxidation (Schreurs et al., 1997), compared to PBAA, as NBAA are absorbed rapidly. Furthermore, studies in rats have shown that the catabolism of non-bound leucine surpasses that of protein-bound leucine (albumen) (Bujko et al., 2007; Nolles et al., 2009).

The intravenous LD_50_ of ammonium acetate in broilers, at 2.72 mmol/kg, is roughly half that observed in mice (5.64 mmol/kg), as reported by Wilson et al. (1968). This highlights the necessity for broilers to rapidly detoxify any increases in plasma NH_3_ concentrations. The detoxification of NH_3_ is predominantly accomplished by its condensation with glutamic acid to produce glutamine, a reaction facilitated by the enzyme glutamine synthetase (Hakvoort et al., 2017). Glutamine then participates in the Krebs uric acid cycle, where it is transformed into uric acid, a process that necessitates 1 mol of glycine for each mole of uric acid excreted (Salway, 2018). The additional requirement for glycine and serine (or glycine equivalents) in reduced-CP diets to facilitate NH_3_ detoxification has been recognised for some time (Dean et al., 2006). Retrospective estimates from the study by Chrystal et al. (2021), as analysed by Selle et al., 2021), suggest that between 25.0% and 80.9% of dietary glycine was allocated to the krebs uric acid cycle, excluding any contribution from glycine biosynthesis. Furthermore, NH_3_ detoxification imposes metabolic costs, including energy expenditures. In poultry, the production and excretion of uric acid for the elimination of NH_3_–N require a minimal energy expenditure of 64.7 kJ/g of nitrogen excreted as uric acid (van Milgen, 2021).

The impacts of higher NH_3_ plasma levels on insulin in mammalian species and from in vitro studies are of some relevance. For example, Feldman and Lebovitz (1971) reported that NH_4_^+^ depresses insulin secretion from isolated pancreatic tissue taken from golden hamsters. Both glucose disappearance rates and insulin secretions were decreased proportionately in normal Wistar rats with elevated plasma NH_3_ levels induced by ammonium acetate infusions in Schlienger et al. (1975). Also, Sener et al. (1978) reported that NH_3_ caused a dose-related, rapid, and reversible inhibition of glucose-stimulated insulin release in isolated pancreatic cells from rats. After the administration of low concentrations of ammonium chloride to Sprague–Dawley rats, insulin failed to stimulate protein synthesis in hepatocytes (Bessman et al., 1991). Finally, Schlienger and Imler (1978) suggested that elevated NH_3_ plasma levels may increase insulin resistance in rats. Thus, there is the possibility that elevated NH_3_ plasma levels may depress pancreatic insulin secretion and/or increase insulin resistance in broiler chickens. If this is the case, without the anabolic impacts of insulin, net protein synthesis would be compromised.

### 3.5 Metabolic acidosis

Low-grade metabolic acidosis was described as a driver of insulin resistance in humans by DiNicolantonio and O'Keefe (2021). There are indications that dietary CP reductions may result in metabolic acidosis and the metabolism of NH_3_ plays a critical role in acid-base homeostasis (Weiner and Verlander, 2017). The catabolism of amino acids in broilers offered reduced-CP diets, particularly methionine, cysteine and cationic amino acids, has an acidifying impact as they generate endogenous acid (H^+^) production (Poupin et al., 2012). Increased lysine HCl inclusions, which are typical in reduced-CP diets, have an acidifying effect as one gram of dietary L-lysine·HCl increases dietary acid levels by 7 mEq/kg (Patience, 1990). In fact, L-lysine·HCl has been used to treat metabolic alkalosis in human patients (Bondoli et al., 1980). Metabolic acidosis, induced by ammonium chloride in humans, was shown to increase protein breakdown and amino acid oxidation by Reaich et al. (1992). These researchers concluded that acidosis is a catabolic factor stimulating protein degradation and amino acid oxidation. Moreover, Mitch et al. (1994) reported that metabolic acidosis accelerates protein degradation rates by stimulating the ubiquitin-proteasome pathway in rats.

Acid-base homeostasis and amino acid metabolism are closely intertwined in animal nutrition (Patience, 1990). Normally, glutamine is utilised by the small intestine and liver; however, in the event of metabolic acidosis, glutamine is extracted and catabolised in the kidneys. This generates NH_4_^+^ ions, which are excreted in the urine to facilitate the excretion of acids and HCO_3_^-^ ions. The renal extraction of glutamine is balanced by an increased release from muscle and liver and by a decreased utilisation in intestinal mucosa. Thus, glutamine is pivotal to acid-base homeostasis as metabolic acidosis is compensated by a net release of HCO_3_^-^ arising from the renal metabolism of glutamine (Taylor and Curthoys, 2004). It was suggested by Rennie et al. (1989) that glutamine-sodium cotransporters in muscle membranes regulate the intracellular glutamine pool and are involved in the promotion of protein synthesis by glutamine and Wu and Thompson (1990) found that glutamine had an overall anabolic effect in isolated avian skeletal myocytes. It was concluded that additions of glutamine and asparagine to broiler diets permitted higher NBAA inclusions before growth performance or nitrogen accretion was compromised in Ibrahim et al., 2024a, Ibrahim et al., 2024b. This was attributed to glutamine and asparagine attenuating challenges to acid-base homeostasis via a compensation of metabolic acidosis. Metabolic acid loads have been associated with impaired glucose homeostasis and insulin resistance as metabolic acidosis interferes with intracellular insulin signaling pathways (Guardia et al., 2018) and Hayata et al. (2014) found that low extracellular pH is involved in insulin resistance in skeletal muscle.

The dietary electrolyte balance (DEB) of soybean meal (478.6 mEq/kg) comfortably exceeds that of maize (71.5 mEq/kg) across 10 samples of each feedstuff, as reported by Sumanthi et al. (2015). Thus, DEB will decline from dietary CP reductions without appropriate adjustments and Waldroup (2007) suggested this may be a contributing factor to the poor performance of birds offered reduced-CP diets. In Chrystal et al. (2020), the possibility of this was investigated, which compared a 156 g/kg CP, maize-based with DEB of either 230 or 120 mEq/kg, but this difference did not impact growth performance. However, the lower DEB of 120 mEq/kg did significantly depress apparent jejunal digestibilities of six amino acids (arginine, isoleucine, leucine, methionine, valine, proline) by 3.40% (0.766 vs. 0.793) on average. Classically, concentrations of sodium, potassium and chloride in diets determine dietary electrolyte balance (DEB [mEq/kg] = Na^+^ + K^+^ Cl^−^); however, this basic equation may not identify DEB with sufficient precision and such reservations were expressed by Johnson and Karunajeewa (1985). There researchers recommended a DEB between 250 and 300 mEq/kg in standard starter (220 g/kg CP) and finisher (185 g/kg) broiler diets. This suggests that the impacts of a far higher DEB values should be evaluated in reduced-CP diets as it is evident that dietary CP reductions have the potential to lower DEB which could, in turn, promote metabolic acidosis (Mushtaq and Pasha, 2013).

### 3.6 Feathering

Feathers have a protein (N) content of 957 g/kg (Stilborn et al., 1997). T amino acid requirements for feathering constitute an outright loss of amino acids, although this attrition is inevitable. This is because mature feathers lack any blood supply; thus, moulting and shedding of feathers represents an effective loss of constituent amino acids (Vargas et al., 2020). Nearly 50% of feather protein is composed of five amino acids: serine (11.4%), glutamic acid (10.6%), proline (9.67%), leucine (8.20%) and cysteine (7.33%). The amino acid profiles relative to lysine in feathers and breast muscle (Hamm, 1981) in comparison to ideal acid ratios are also displayed in Table 1. Clearly, the amino acid constituencies of feathers and breast muscle are radically different and both differ from the ‘ideal’ profile of dietary amino acids.

**Table 1:** Amino acid profiles of feathers, breast muscle and ideal dietary amino acid ratios in diets for broiler chickens from 21 to 42 d post–hatch.

Amino acid	Feathers^1^		Breast muscle^2^		Ideal amino acid ratios^3^
	Concentration, g/kg	Relative to lysine	Concentration, g/kg	Relative to lysine	
Arginine	67.3	354	69.8	84	103
Histidine	6.1	32	42.8	52	37
Isoleucine	46.4	244	43.4	52	70
Leucine	79.8	420	82.5	99	118
Lysine	19.0	100	83.0	100	100
Methionine	5.5	29	32.5	39	44
Phenylalanine	46.5	245	40.3	49	68
Threonine	43.5	229	47.7	57	69
Tryptophan	5.4	28	–	–	17
Valine	68.1	358	46.9	57	79
Alanine	44.3	233	57.8	70	88
Aspartic acid	62.2	327	98.0	118	136
Cysteine	71.3	375	7.0	8	29
Glutamic acid	103.1	543	149.1	180	289
Glycine	69.5	366	45.6	55	119
Proline	94.1	495	40.5	49	137
Serine	113.8	599	49.3	59	72
Tyrosine	27.0	142	39.5	48	45
Total	972.9		975.7		

1 Adler et al. (2018); Greenhalgh et al. (2020b); Wecke et al. (2018); Stilborn et al. (1997).

2 Hamm (1981).

3 Maharjan et al. (2021); Wu (2014).

Four decades ago, feathers represented 18.9% of a broiler's body weight (Crawley et al., 1980). However, a current finding indicates that feathers represent an average of 3.43% of body weight in broiler chickens at 56 d post–hatch (Vargas et al., 2020). Also, it has been estimated that 10% of protein intake per day is required for feather production up to 42 d post–hatch in broilers (Van Emous and Van Krimpen, 2019). The proportion of six dietary amino acids deposited in feathers was estimated by Leeson (2021) to range from cysteine (52%) to valine (18%), arginine (14%), threonine (8%), lysine (5%) and methionine (5%) from 24 to 39 d post–hatch. Consequently, the partitioning of amino acids towards either feather protein or skeletal muscular protein assumes importance. In the event of dietary amino acid inadequacies, it has been asserted that feather protein synthesis is given priority in broilers (Conde-Aguilera et al., 2016; Wecke et al., 2018). Methionine and cysteine were utilised more efficiently for feathering than for feather-free body protein deposition in Pacheco et al. (2020). Moreover, Wylie et al. (2003) concluded that amino acids were preferentially partitioned towards feathering rather than whole-body protein synthesis in turkeys.

Importantly, Leeson and Walsh (2004) highlighted that the optimal amino acid balance required for feather development, independent of traits such as growth and feed efficiency, may not have been accurately established. Notably, Greenhalgh et al. (2020b) reported poor feathering in broilers fed wheat-based, reduced-CP diets. Shift of 215 to 162.5 g/kg CP in diets significantly reduced feather scores (3.7 to 1.8) and severely impacted growth performance. The dietary-CP declines in analysed dietary concentrations of three of the dominant amino acids in feathers pursuant to the dietary-CP reductions also led to notable decreases in the concentrations of three key amino acids essential for feather composition, which are serine, glutamic acid and proline by 37.6%, 31.6%, and 29.7%, respectively. These amino acids were not supplemented as NBAA in the reduced-CP diets, which likely contributed to the observed outcomes.

Instructively, Taruscio and Murphy (1995) compared 3-methylhistidine excretion in moulting or non-moulting sparrows. Excretion of 3-methylhistidine in moulting sparrows was significantly higher by 55.3% (1.235 vs. 0.795 μmol/d) than non-moulting sparrows over two assessments. One possible interpretation is that protein turnover was increased to compensate for the inevitable loss of amino acids from moulting. Nevertheless, the unorthodox amino acid demands for feathering in poultry is a complicating factor in respect of both protein turnover and identifying dietary amino acid requirements. It is evident that feathering merits closer attention in amino acid utilization in broiler chickens, especially in reduced-CP diets.

### 3.7 Breast meat yields

Breast meat yields are indicative of protein synthesis and are pivotally important to the profitability of the chicken-meat industry. Protein (N) contents of breast meat in male and female chickens are 199 and 216 g/kg, respectively (Hailemariam et al., 2022), and their amino acid profiles have been recorded by Hamm (1981). Breast meat yields in relation to dietary CP reductions in wheat- and sorghum-based diets from four studies completed at this institution are shown in Table 2. A reduction of 34 g/kg in dietary crude protein, from 205 to 171 g/kg, offered to birds between 14- and 35-d post–hatch, resulted in a 6.67% decrease in breast meat yield (210 g/kg compared to 225 g/kg). The linear relationship (*r* = 0.493; *P* = 0.074) of dietary CP and breast meat yields approached significance across the tabulated data and, interestingly, increasing digestible protein (N) intakes enhanced breast meat yields quadratically (*r* = 0.799; *P* = 0.004). These outcomes indicate that dietary CP reductions depress net protein synthesis in breast muscle, either by retarding protein deposition and/or accelerating protein degradation, where the second factor may the more influential.

**Table 2:** The impacts of dietary crude protein (CP) reductions of broiler diets on protein (N) intakes, digestible protein (N) intakes, weight gains and breast meat yields in four studies.

Feed grain	Feeding Period, d	Dietary CP, g/kg	Protein (N) intake, g/bird	Digestible protein (N) intake, g/bird	Weight gain, g/bird	Breast meat yield, g/kg
Wheat^1^	14–35	205	640	540	2252	239
		170	520	447	2024	218
Wheat^2^	15–36	211	647	549	2771	240
		173	480	392	1880	222
Wheat^3^	14–35	201	580	435	1942	205
		175	493	335	1781	191
		209	600	466	1986	206
		161	447	337	1703	185
Wheat^4^	14–35	198	621	494	2184	229
		167	517	412	1964	231
Sorghum^1^	14–35	204	651	540	2332	225
		168	518	433	2147	210
Sorghum^4^	14–35	205	631	478	2122	232
		180	552	401	2096	216
**Mean values**						
High CP		205	624.3	500.3	2227	225
Low CP		171	503.9	393.9	1942	210

1 Macelline et al. (2023a).

2 Macelline et al. (2023b).

3 Macelline et al. (2023c).

4 Wang et al. (2025).

## 4 Future directions

It is evident in the foregoing that there is the opportunity to attenuate the attrition of amino acids during the life span of broiler chickens. The enhancement of amino acid digestibilities is one, relatively straightforward, step. The possibility of manipulating the ‘catabolic ratio’ between glucose and amino acids in the gut mucosa in broiler chickens certainly merits further investigation as if this objective can be achieved it should be highly rewarding. As it stands, our knowledge of the entries of amino acids into anabolic and catabolic pathways within enterocytes is rudimentary. Our understanding of the anabolic role of insulin and the regulation of protein turnover in broiler chickens, again, obviously merits further investigation. It appears that any strategy that attenuates protein degradation would reduce extents of amino acid attrition.

### 4.1 Amino acid digestibilities

Exogenous phytases have the capacity to enhance amino acid digestibilities as demonstrated by Amerah et al. (2014) and Truong et al. (2015). Amerah et al. (2014) reported that phytase at 1000 FTU/kg improved the mean apparent ileal digestibility coefficients of 17 amino acids by 13.2% (0.728 vs. 0.824). Truong et al. (2015) reported that 500 FTU/kg phytase increased mean apparent ileal digestibility coefficients of 16 amino acids by 7.24% (0.904 vs. 0.843). However, in the proximal jejunum, phytase increased mean amino acid digestibility coefficients by a remarkable 49.7% (0.720 vs. 0.480). The probable genesis of this capacity of phytase to increase amino acid digestibilities is that this feed enzyme both enhances protein digestion in the small intestine and facilitates absorption of amino acids (Selle et al., 2023a, 2023b). This is because binary protein-phytate complexes are refractory to pepsin digestion but phytase essentially prevents their formation by the prior hydrolysis of phytate. Also, phytase has a profound sodium sparing effect (Selle et al., 2009) and 1000 FTU/kg phytase activity has been shown to increase the jejunal density of "sodium pumps" by 18.4% (13.59 vs. 11.48 μmol/mg Na^+^K^+^-ATPase) in broiler chickens (Liu et al., 2008). Importantly, sodium pump activity along the small intestine is largely dependent on cytoplasmic Na concentrations within enterocytes (Therien and Blostein, 2000). Thus, the sodium sparing effect of phytase directly facilitates intestinal uptakes of monomeric amino acids by sodium-dependent transport systems and indirectly facilitates intestinal uptakes of oligopeptides via PepT-1 and NHE in tandem to have positive impacts on intestinal uptakes of both oligopeptides and monomeric amino acids. That said, exogeneous phytases are routinely included in broiler diets globally, very often at elevated inclusion levels.

### 4.2 Catabolic ratio of amino acids and glucose in the gut mucosa

The utilisation of amino acids for energy production certainly represents an attrition of amino acids, but it may be possible to minimise these losses by increasing the utilisation of glucose as an energy substrate. As mentioned, there are suggestions (Weurding et al., 2003; Enting et al., 2005) that the dietary provision of slowly digestible starch and the prolonged availability of glucose as an alternative energy substrate along the small intestine could spare amino acids from catabolism in enterocytes. This aspect merits further investigation and the sequential, post-prandial determinations of glucose concentrations in plasma taken from the anterior mesenteric vein may prove instructive. Van der Meulen et al. (1997) determined the effect of starch source on the net portal flux of glucose and amino acids in the pig and significant differences between starch sources (maize versus pea) were observed. However, these determinations required catheterization of the mesenteric vein and artery and the portal vein, which would not appear to be practical in broiler chickens. Nevertheless, it may prove instructive to investigate glucose, insulin and glucagon post prandial plasma concentrations in the anterior mesenteric vein as influenced by different dietary starch sources in poultry.

### 4.3 L-carnitine and protein turnover

L-carnitine was included at 0, 75 and 150 mg/kg in sorghum-based broiler diets containing 220, 190 and 160 g/kg of CP in Greenhalgh et al. (2022). The premise was that L-carnitine would decrease fat deposition as Xu et al. (2003) reported that 50 mg/kg L-carnitine supported reduction of 16.2% relative abdominal fat-pad weights. However, L-carnitine did not significantly alter relative fat-pad weights (*P* > 0.65) in the Greenhalgh et al. (2022) study. A addition of 150 mg/kg L-carnitine to 220 g/kg CP diets significantly depressed weight gains by 3.37% (2150 vs. 2225 g/bird) from 7 to 33 d post–hatch. In complete contrast, 150 mg/kg L-carnitine in 160 g/kg CP diets significantly increased weight gains by 15.9% (1593 vs. 1374 g/bird) and improved FCR by 3.16% (1.564 vs. 1.615). Moreover, 75 mg/kg L-carnitine in 160 g/kg CP diets significantly improved weight gains by 15.0% (1580 vs. 1374 g/bird) and FCR by 5.82% (1.521 vs. 1.615). The researchers proposed that the growth performance improvements associated with L-carnitine supplementation in broiler chickens fed 160 g/kg CP diets might be linked to its ability to mitigate the effects of NH_3_ intoxication. Notably, L-carnitine has been shown to offer protective effects against NH_3_ toxicity (Kloiber et al., 1988) and, while this assumption may be valid, plasma NH_3_ concentrations were not determined in Greenhalgh et al. (2022).

L-carnitine is a quaternary amine (β-hydroxy γ-trimethylaminobutyrate), which may be synthesised from methionine and lysine in the liver and is present in feedstuffs to varying extents. According to Arslan (2006), soybean meal contains 15 g/kg and sorghum and wheat 5 g/kg L-carnitine. On this basis, dietary L-carnitine levels declined from 7.33 to 6.05 and 4.65 g/kg as dietary CP levels were reduced in Greenhalgh et al. (2022). The functional effects of L-carnitine in poultry nutrition have been reviewed by Adabi et al. (2011). Given the hepatic biosynthesis, L-carnitine is not usually considered to be an essential nutrient but it may become limiting when diets are low in L-carnitine content or rich in fat and under conditions of stress or high performance (Murali et al., 2015). Interestingly, Rodehutscord et al. (2002) reported that an 80 mg/kg L-carnitine inclusion in broiler diets containing 80 g/kg fat improved weight gain by 5.73% (756 versus 715 g/bird) and FCR by 4.86% (1.37 versus 1.44) in a 16-d growth trial. On dry matter basis, the control diet contained an analysed concentration of 3.9 g/kg L-carnitine.

Clearly, L-carnitine is a multi-functional molecule; however, importantly, there are indications that L-carnitine suppresses protein degradation. Sprague–Dawley rats were offered either a control diet with a low inherent concentration of L-carnitine or a diet supplemented with 1250 mg/kg L-carnitine in Keller et al. (2013). L-carnitine noticeably depressed concentrations of ubiquitinated proteins in skeletal muscle indicating a diminished degradation of myofibrillar proteins by the ubiquitin-proteasome pathway. This was associated with L-carnitine decreasing the mRNA expression and concentrations of MuRF1 in muscle. The muscle-specific RING finger protein 1, or MuRF1, has been described by as a "master regulator of muscle mass" and is believed to play important roles during skeletal muscle atrophy (Peris-Moreno et al., 2020). In addition, dietary L-carnitine has been shown to influence gene expression in skeletal muscle of young pigs by Keller et al. (2011). These researchers concluded that L-carnitine may have beneficial effects on skeletal muscle mass through stimulating the anabolic IGF-1 pathway and suppressing pro-apoptotic and atrophy-related genes. Also, isovaleryl-L-carnitine, a carnitine derivative, has been shown to inhibit proteolysis in perfused rat hepatic tissue (Miotto et al., 1989). Therefore, it looks feasible that the promising growth performance responses generated by L-carnitine in reduced-CP diets in Greenhalgh et al. (2022) was partially due to suppressed protein degradation rates and enhanced net protein synthesis.

### 4.4 Methylhistidine

That the quantitative excretion of 3-methylhistidine in urine provides a reliable index of the rate of myofibrillar protein breakdown was proposed by Munro and Young (1978). The rationale is that the majority of 3-methylhistidine in the body is present in actin and myosin of skeletal muscle, and when these proteins are degraded, 3-methylhistidine is released and excreted unchanged in the urine. Consequently, several studies have assessed protein degradation in broiler chickens based on 3-methylhistidine excretion including Hayashi et al. (1985). These researchers found that average degradation rates of myofibrillar proteins in male layer chicks exceeded those of male broiler chicks by a 1.5-fold factor (4.3 vs. 2.9%/d) at 21, 42 and 63 d post–hatch. They concluded that the relatively rapid growth of broiler chickens stemmed from slower protein degradation rates. Jones et al. (1986) found that 84% of 3-methylhistidine in chickens was derived from muscle and the fractional protein breakdown rate was 5.3% per day, determined from 3-methylhistidine excretion. Subsequently, Tomas et al. (1988) reported that reductions in *N*^*r*^-methylhistidine excretion was related (*P* < 0.001) to FCR improvements in a quadratic manner in broiler chickens selected for increased growth rate, increased feed consumption and enhanced FCR.

However, Rennie and Millward (1983) had contended that the urinary excretion of 3-methylhistidine was a poor indicator of skeletal muscle protein breakdown in mammalian species. More specifically, Harris et al. (1987) concluded that 3-methylhistidine excretion in poultry should not be taken as a reliable index of in vivo muscle protein breakdown. Nevertheless, analyses of 3-methylhistidine plasma concentrations, rather than in excreta, may be a better approach (Sugawara et al., 2009). Plasma concentrations of 3-methylhistidine can be determined by liquid chromatography-tandem mass spectrometry, as described by Shiraishi et al. (2023). It appears that 3-methylhistidine has yet to be used in practical, reduced-CP feeding studies with broiler chickens to determine protein degradation rates. However, Hocking and Saunderson (1992) reported that transition of CP from 150 to 100 g/kg in poultry diets increased 3-methylhistidine excretion by 15.7% (54.4 vs. 47.0 μmol/d), which suggests that dietary CP reductions trigger increases in protein degradation rates. Thus, the incorporation of 3-methylhistidine into appropriately designed reduced-CP feeding studies with analyses of 3-methylhistidine plasma concentrations could prove highly instructive.

### 4.5 Protected amino acids

A strategy that merits further investigation is the application of protected or “delayed release” amino acids with retarded intestinal uptake rates more closely aligned with protein-bound amino acids; a possibility raised by Selle et al. (2022c). Advanced oral delivery technologies for the delayed absorption of amino acids and peptide molecules have been developed (Choonara et al., 2014). Microencapsulated lysine has shown some promise in grower-finisher pigs (Prandini et al., 2013). Indeed, it has been reported that lipid-encapsulated lysine and methionine were more effectively utilised in broiler chickens than non-bound entities (Sun et al., 2020). These researchers concluded that supplemental levels of synthetic lysine and DL-methionine may be decreased by approximately 20% in the encapsulated form without adversely affecting growth performance of broiler chickens. Another possibility is the evaluation of peptides or protein isolates/concentrates. This may be a more economically feasible and effective approach to supplement reduced-CP diets with amino acids than an array of monomeric non-bound amino acids.

## 5 Conclusions

From the foregoing it is evident that the attrition of amino acids in the life cycle of broiler chickens is quite extensive, but the extent to which it can be attenuated is problematic. Certainly, more fundamental explorations into the metabolic fates of amino acids and starch/glucose in the small intestinal gut mucosa are required but will be challenging. Further investigations into the possibility that starch from slowly digestible feedstuffs reduce amino acids catabolism within enterocytes should be completed. It would be a real breakthrough if it can be demonstrated that the amino acid-glucose catabolic ratios in the gut mucosa are subject to favourable manipulation by dietary strategies. Again, more fundamental explorations into protein turnover in broiler chickens are required, but the area is complex. However, if it can be shown that the determination of 3-methylhistidine concentrations in systemic plasma is a sufficiently accurate biomarker for protein degradation, research into this complex area would be greatly facilitated. Experiments involving both L-carnitine and 3-methylhistidine in broiler chickens could prove highly instructive, as L-carnitine may have the capacity to depress protein degradation and positively impact protein turnover. The role of insulin in avian species will remain a conundrum without further research, particularly the regulatory role of insulin in relation to protein turnover as broilers mature and the various influential factors including metabolic acidosis and dietary electrolyte balance. Finally, nutritionists should continue to strive to formulate diets for broiler chickens that faithfully and accurately meet amino acid requirements, which will be challenged by any adoption of reduced-CP diets with increased levels of NBAAs. To the extent that this elusive objective can be realised, circulating NH_3_ levels arising from post-enteral amino acid imbalances will be restricted to the advantage of broiler growth performance.

## Credit Author Statement

**Shemil P. Macelline:** Writing – review & editing, Data curation, Writing – original draft, Conceptualization, Resources. **Sonia Y. Liu:** Validation, Writing – review & editing, Supervision, Writing – original draft, Conceptualization. **Peter H. Selle:** Supervision, Conceptualization, Writing – review & editing, Formal analysis, Writing – original draft, Data curation.

## Declaration of competing interest

We declare that we have no financial and personal relationships with other people or organizations that can inappropriately influence our work, and there is no professional or other personal interest of any nature or kind in any product, service and/or company that could be construed as influencing the content of this paper.
